# The CCN family of proteins: structure–function relationships

**DOI:** 10.1016/j.tibs.2008.07.006

**Published:** 2008-10

**Authors:** Kenneth P. Holbourn, K. Ravi Acharya, Bernard Perbal

**Affiliations:** 1 Department of Biology and Biochemistry, University of Bath, Claverton Down, Bath BA2 7AY, UK; 2 Department of Dermatology, University of Michigan, Ann Arbor, MI 48109, USA; 3 Present address: Research and Development, L’Oréal USA, 111 Terminal Avenue, Clark, NJ 07066, USA

## Abstract

The CCN proteins are key signalling and regulatory molecules involved in many vital biological functions, including cell proliferation, angiogenesis, tumourigenesis and wound healing. How these proteins influence such a range of functions remains incompletely understood but is probably related to their discrete modular nature and a complex array of intra- and inter-molecular interactions with a variety of regulatory proteins and ligands. Although certain aspects of their biology can be attributed to the four individual modules that constitute the CCN proteins, recent results suggest that some of their biological functions require cooperation between modules. Indeed, the modular structure of CCN proteins provides important insight into their structure–function relationships.

## The CCN family of proteins

The CCN family of proteins is a complex family of multifunctional proteins containing six members designated CCN1 to CCN6. The CCN acronym was introduced from the names of the first three members of the family to be discovered: Cyr61 (cysteine-rich protein 61), CTGF (connective tissue growth factor) and NOV (nephroblastoma overexpressed gene) [1]. Owing to the multifunctional nature of these proteins, they have been identified in several other biological studies and have been assigned multiple names; however, in this review we will use the official nomenclature [2] (Table 1). (See also http://ccnsociety.com). This family of secreted extracellular matrix (ECM)-associated proteins is involved in a wide range of important functional pathways, including adhesion, mitogenesis, migration and chemotaxis, cell survival, differentiation, angiogenesis, chondrogenesis, tumourigenesis and wound healing. They have also been implicated in many human diseases (for some comprehensive reviews, see Refs [3–5]).

**Figure 1:**
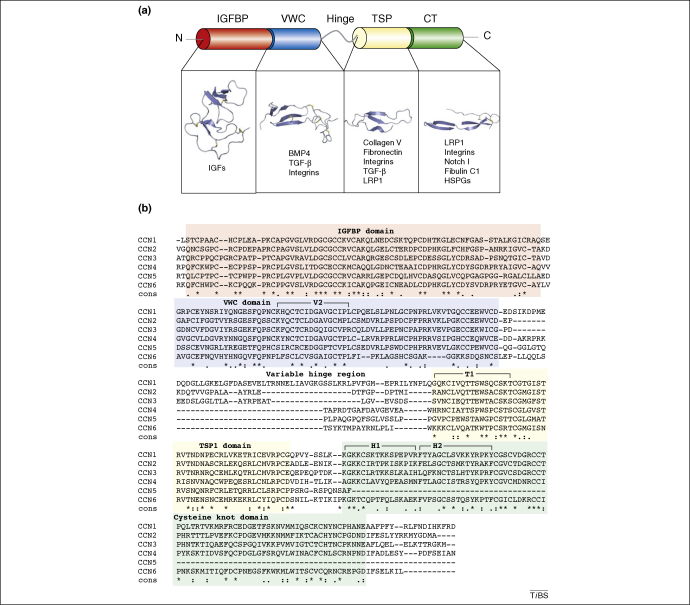
Arrangement of CCN domains. **(a)** A diagram showing the signal peptide (SP), insulin-like growth factor binding domain (IGFBP) in red, von Willebrand factor C repeat (VWC) in blue, thrombospondin type-1 repeat (TSP-1) in yellow and cysteine knot (CT) in green. The protein is split into two halves separated by a variable ‘hinge’ region. Some of the known binding partners of each module are also listed: insulin-like growth factors (IGFs); bone morphogenic protein 4 (BMP4); transforming growth factor β (TGF-β); LDL receptor protein 1 (LRP-1); and heparin sulphated proteoglycans (HSPGs). **(b)** A sequence alignment of the CCN protein family. The sections of the sequence corresponding to each domain are shaded according to the colour scheme used in (a). The asterisks highlight the conserved residues and include the 38 cysteines that form part of the key motifs of each domain. The three regions of the sequence that have been implicated directly in integrin binding are also highlighted. These areas are highlighted in bold text. The V2 site binds integrin αvβ3 [104]; the T1 site binds α6β1 [105]; the H1 site also binds α6β1; and the H2 site binds HSPGs [106]. The alignment was constructed by the T-Coffee server [107].

The unifying feature of this family of proteins is their mosaic structure, consisting of discrete modules that share identity with functional domains of other regulatory molecules. A prototypical CCN protein contains an N-terminal secretory signal peptide and four functional domains: (i) an insulin-like growth factor binding protein-like module (IGFBP); (ii) a von Willebrand factor type C repeat module (VWC); (iii) a thrombospondin type-1 repeat module (TSP-1); and (iv) a cysteine-knot-containing module (CT) (Figure 1). Apart from CCN5, which lacks a CT module, all CCN proteins exhibit the same type of organization and share a closely related primary structure, which includes a series of 38 cysteine residues that are strictly conserved in position and number. However, CCN6 lacks four cysteine residues in domain II (VWC).

The 38 cysteine residues that spread across the four modules represent almost 10% of the CCN molecule by mass. A short sequence that varies greatly in both composition and length among the CCN family members is located directly after the VWC domain. It was proposed to act as a hinge between the first and second half of the molecule [1,3]. Recent data have established that each of the linker regions that separate the four CCN modules is susceptible to proteolysis [6]. Cleavage at these sites might be responsible for the production of truncated molecules and individual modules [4] that display distinct biological properties. Moreover, these events might constitute an additional process to regulate the biological activity of the CCN proteins [5,7].

The six CCN family members share ∼30–50% identity in their primary structure (∼40–60% similarity) [6,8]. In addition to their highly conserved sequence alignment (Figure 1), the CCN proteins share a common intron/exon pattern, and each CCN family member contains five exons. The first exon corresponds to the signal sequence, and the remaining four exons correspond to one of the discrete protein modules. This exon arrangement suggests a certain amount of ‘evolutionary shuffling’ of functional domains that is also seen in other matricellular modular proteins [1,6,9].

Although some CCN protein functions are directly related to an individual functional module, many functions are thought to result from modules acting in concert. Truncated CCN2 molecules, each comprising two modules, can function independently to stimulate differentiation or proliferation of fibroblast and to increase collagen synthesis [10]. Production of truncated proteins showing altered biological properties, or proteins without some internal modules, has been associated with specific developmental stages and pathological situations [11].

The make-up of a single protein into four modules that share identity with four large classes of regulatory proteins, which play fundamental roles in cellular biology, makes the CCN family members unique mosaic proteins. The pleiotropic functions of the CCN proteins stem from their tetramodular structure. However, a better knowledge of the physical organization of the CCN proteins is required to understand their crucial functions in both normal and pathological conditions. This review summarizes the structural features of CCN proteins as they relate to their diverse functions.

## What are the biological roles of the CCN proteins?

The roles of CCN proteins as key regulatory ECM components and as signalling molecules involved in important biological functions have been covered in great detail in other reviews [3,11]. The following sections will focus on the biological properties of CCN proteins that appear to be either common or specific to individual members of the CCN family, to highlight possible relationships between their biological function(s) and particular structural features.

### Adhesion and extracellular matrix remodelling

Adhesion, signalling and migration are processes in which the CCN proteins might be involved, owing to the connection of CCN proteins to the ECM. CCN1 and CCN2 both mediate adhesion in several types of cell [6]. In addition, CCN2 is required for adhesion and ECM contraction [12]. Recently, it was reported that CCN3 activity increases the adhesion of normal melanocytes to collagen type IV and controls discoidin domain receptor 1 (DDR1)-mediated three-dimensional localization of melanocytes in skin [13].

The ability of CCN proteins to promote adhesion involves cell survival because interactions with several surface integrins and transforming growth factor β (TGF-β) can prevent cell apoptosis [6,14]. In addition to interacting directly with ECM components, including fibronectin and heparin [6,12], CCN1 and CCN2 can induce ECM synthesis and TGF-β-mediated fibronectin and collagen expression via the induction of the SMAD (see Glossary) family of transcription factors [6]. However, recent data indicate that CCN2-dependent stimulation of collagen and extracellular matrix synthesis in murine embryonic fibroblasts does not involve the SMAD signalling pathway [12,15].

Recombinant CCN3 induces adhesion of vascular smooth muscle cells, endothelial cells and fibroblasts through interactions with integrin cell surface receptors and heparin sulphate proteoglycans [12]. Despite the absence of an Arg-Gly-Asp (RGD) tripeptide cell adhesion motif, which is often required for physical interaction with integrins, CCN3 associates with the vitronectin receptor αvβ3, integrins α5β1 and α6β1, possibly through its C-terminal domain, and with integrins αvβ3, αvβ5, α2β1, α3β1 and α7β1, which is consistent with the involvement of a cell-type-dependent subset of adhesion molecules.

### Skeletal development and chondrogenesis

Because integrins and other adhesive signalling molecules perform vital roles in tissue remodelling [16], it is not surprising that the CCN proteins are also involved in skeletal formation and development [6,17]. All CCN family members are involved in bone cell development and differentiation (both osteoblasts and chondrocytes). Indeed, their expression increases during fractures or breaks, although the different family members appear to perform distinct functions or are activated by different growth factors [18]. In osteoblasts, for example, TGF-β signalling increases CCN1, CCN2 and CCN5 expression but decreases CCN4 expression [18]. CCN1, CCN2, CCN3 and CCN6 each induce the expression of chondrogenic markers [12,17]. Recent studies performed using CCN2-deficient cells have also provided new insights into the roles that CCN2 might play in chondrogenesis [19]. In addition to their chondrogenic functions, CCN1 and CCN2 might also participate in cartilage neovascularization through their involvement in angiogenesis [3].

### Angiogenesis and wound repair

The growth of new blood vessels through angiogenesis is important in tissue and cell development, tumour formation and wound healing [6]. The CCN family members are important angiogenic modulators owing to their interactions with several growth factors and integrins [17]. In particular, CCN1 and CCN2, the expression of which is probably induced by growth factors, including fibroblast growth factor (FGF) and TGF-β, mediate angiogenesis through interactions with angiogenic integrins αvβ3 and α6β1 [3,6,20]. However, in keeping with their status as complex functional modulators, CCN proteins can promote or suppress angiogenesis depending on their specific interactions with growth factors. For example, CCN2 can promote angiogenesis through TGF-β-induced integrin action [3], or it can suppress angiogenesis through interactions with vascular endothelial growth factor (VEGF). Notably, VEGF-dependent suppression can be alleviated after matrix metalloprotease-mediated CCN2 cleavage [12].

Although the angiogenic effects of CCN1, CCN2 and CCN3 are beginning to be understood, the roles that CCN4, CCN5 and CCN6 play in angiogenesis remain less clear [17]. Differences in gene expression might play a role in the distinct physiological roles of the CCN molecules: CCN1 probably functions during early embryonic development; CCN2 probably has a role in developmental processes that occur later in life, including tissue regeneration; and CCN3 probably plays a supporting role. CCN1, CCN2 and CCN3 are induced during adult tissue repair [12]. The contribution of CCN2 in scarring and the fibrotic response has been reviewed in detail [12].

The role of CCN proteins in wound healing is probably similar to the role they play in angiogenesis. The important events in wound healing, for example chemotaxis, vascularization, mitogenesis of fibroblasts, angiogenesis, adhesion and ECM manipulation, are all regulated, in part, by the CCN family. CCN1 and CCN2 are upregulated during cutaneous wound healing [3,14], and CCN1 expression also increases during liver regeneration [3,21]. By contrast, in a mouse model of cutaneous wound healing, CCN3 transcripts are rapidly reduced during the first three days after wound formation or re-epithelialization [20] when keratinocytes grow and migrate. Interestingly, CCN3 production is induced in the epidermis and dermis 5 to 7 days after injury, when keratinocytes and fibroblasts undergo redifferentiation to restore barrier function and the dermal matrix, respectively [22].

## Proliferation

Regulation of cell proliferation was one of the first biological functions assigned to CCN proteins. Indeed, CCN1 and CCN2 were initially described as proteins encoded by an immediate early gene and a cysteine-rich mitogen, respectively [3]. By contrast, it was initially proposed that CCN3 acted as a growth inhibitor [11]. Over the past decade, a wealth of experimental data has confirmed that CCN1 and CCN2 activity stimulates cell proliferation. However, as recombinant proteins, they do not affect cell proliferation, nor can they potentiate the stimulatory effects of other cytokines (for comprehensive reviews see Refs [6,7,23]).

Apart from the antiproliferative effects of CCN3 that have recently been documented at a mechanistic level [24], very little is known regarding CCN protein signalling pathways and targets that might account for their effects on cell proliferation. Indeed, despite the many examples that established a relationship between CCN2 expression and growth stimulation, including its involvement of p44/42 mitogen-activated protein kinase (MAPK)/extracellular-signal-regulated kinase (ERK) signalling pathway [25], it was reported only recently that *CCN2* knockout mice display reduced cell proliferation and hypoplasia *in vivo* [26]. CCN2 is also required for maximal ERK activation via syndecan 4 [27].

Interestingly, purified CCN2 domains exhibit differential effects on CCN2-dependent proliferative signalling pathways [28]. In human umbilical vascular endothelial cells (HUVECs), the VWC, TSP and CT domains, as well as a full-length CCN2, efficiently activate the ERK signal transduction cascade, whereas the IGFBP domain prominently activates the Jun N-terminal kinase (JNK) pathway. Interestingly, only a mixture of the four CCN2 domains could induce significant p38 MAPK activation in human chondrocytic HCS-2/8 cells, as does the full-length CCN2 protein.

Consistent with its expression being associated with fibroblast quiescence [11], CCN3 elicits an inhibitory effect in normal and pathological conditions, including tumour-derived cells in which high CCN3 levels are detected and associated with higher proliferation rates and/or high tumourigenic potential [7,11,24,29,30]. Although growth inhibitory functions have also been assigned to CCN5 [6] and CCN6 [31], CCN3 remains the best example of a negative regulator of cell growth in the CCN family. Mechanistically, the CCN3 CT module is sufficient to govern inhibition of cell growth [24], although recent data indicate that other domains might be involved in this process [32,33]. An explanation for the growth-inhibitory effect of CCN3 was provided by the observation that CCN3 overexpression triggers an accumulation of cells at the S-phase of the cell cycle [24]. Indeed, [^3^H]thymidine incorporation increases in cells treated with recombinant CCN3 [11]. Likewise, the reduction in CCN3 levels observed immediately after wounding or re-epithelialization (20) is in agreement with the ability of CCN3 to negatively regulate fibroblast proliferation. Interestingly CCN5, which shows growth arrest activity, lacks a CT module.

CCN3-dependent growth inhibition correlates with activation of the cell-cycle inhibitor p21 (Cip1/Waf1) [24,34] and is regulated by cell-density-dependent post-translational processes [22]. The expression of both p21 and CCN3 is regulated by the tumour suppressor p53 [35]. Because CCN1 and CCN2 often exhibit expression patterns opposite to those of CCN3 during development [11] and in cultured cells [30], the regulation of cell proliferation might involve a subtle balance of negative and positive signals induced by different members of the CCN family [5,29].

## Tumourigenesis

Abnormal levels of most CCN family members are associated with tumour development [6]. Recent data [24] suggest that enhanced or reduced expression of CCN proteins in tumours results from deregulated transcriptional and post-transcriptional regulatory processes. Whereas full-length CCN proteins can exhibit anti-proliferative behaviour, truncated CCN isoforms can act as tumourigenic agents [7] and promote tumourigenesis. Similar effects are observed when inappropriate levels of CCN proteins are produced [36]. Indeed, evidence links all CCN family members to tumourigenic effects [5,6,29,37].

CCN1, CCN2 and CCN4 are expressed at elevated levels in advanced breast cancers; increased CCN1 levels are thought to lead to more invasive breast cancers [36]. CCN1 and CCN2 levels are also elevated in pancreatic tumours; CCN2 expression is linked to malignancy. Increased CCN2 levels are also present in some brain tumours [36]. Likewise, CCN4 and CCN6 expression levels are significantly raised in some colon cancers [36]. By contrast, expression levels of CCN1–5 are reduced in high grade chondrosarcomas [4], and CCN3 expression is reduced in brain tumours [4] and chronic myeloid leukaemia [38].

Because CCN3 was the first member of the CCN family to be associated with tumour development [3], it is the most studied CCN protein with regard to tumour typing and prognosis [6]. CCN3 expression is upregulated in prostate and renal carcinomas [7]. Likewise, CCN3 is highly expressed in both visceral and nodal melanoma metastases and it has been proposed that it regulates dissemination of the tumour cells through the interaction of tumour cells with the ECM [39]. Moreover, CCN3 expression, together with connexin 43, correlates positively with reduced tumour cell growth in choriocarcinomas and glioblastomas [6]. By contrast, another study suggests that progression of melanoma is associated with CCN3 downregulation; these differences highlight the complexity of CCN biology in tumours [40].

Interestingly, the expression of CCN proteins can also serve as a prognostic tool in human tumours. For example, molecular evidence has demonstrated that CCN1 and CCN2 contribute to glioma tumour progression. CCN1 and CCN2 expression levels also correlate positively with tumour grade and pathology and are prognostic indicators of patient survival in these tumours [6]. A significant correlation was established between CCN1 and CCN2 expression and survival of patients with lung cancer [41]. In addition, CCN2 expression is an independent prognostic indicator of both tumour recurrence and overall survival for intra-hepatic cholangiocarcinoma [42]. CCN4–6 levels are also of significant prognostic value in breast cancers [43], and reduced CCN6 expression is strongly associated with a poor prognosis in 80% of aggressive inflammatory breast cancers [31]. This lack of CCN6 results in uncontrolled IGF-1-induced cell growth and tumourigenesis. Indeed, the CCN6 IGFBP domain is thought to help regulate IGF-1 availability in healthy tissues [44,45].

In musculoskeletal tumours, including rhabdomyosarcoma, and cartilage tumours, CCN3 expression correlates positively with tumour differentiation. By contrast, in Ewing's sarcoma, CCN3 expression is associated with a higher risk for metastasis [11] and a poor survival prognosis [46,47]. Therefore, the prognostic value of CCN proteins in tumour biology is now opening promising avenues for molecular diagnosis, typing and therapy [30,48].

## The domain structure of the CCN proteins

The variety of biological functions assigned to the CCN proteins is in agreement with the proposed model that CCN proteins are adaptor molecules involved in multimolecular complexes that coordinate and integrate signalling between extracellular ligands and their receptors [7,11]. Therefore, a prediction of CCN protein structures, comparing the spatial organization of their constituent modules, might provide crucial information regarding their pleiotropic functions. The domain structure of the CCN proteins resembles many ECM proteins because they are constructed from a library of commonly used domains [49]. Although the domains have been classified through recognizable motifs, the exact role of each domain in CCN biology is not fully understood. Here, we discuss each domain, focusing on how the predicted three-dimensional structure of the domain might influence function.

### The IGFBP domain

The mammalian IGFBP family consists of six IGFBPs (1–6) that bind IGFs with high affinity (*K*_D_ ∼0.1 nM) to control their transport, localization and metabolic breakdown. The indirect control of IGF function implicates IGFBPs in many important cellular functions, including cell cycle progression, cell proliferation, cell death, cell differentiation, amino acid and glucose uptake, hormone and neurotransmitter secretion, chemotaxis and parts of the immune response [50].

The IGFBP proteins are multidomain proteins with distinct cysteine-rich N- and C-terminal domains linked by a variable linker region. The N-terminal globular domain contains 12 conserved cysteine residues, and the C-terminal domain contains an additional six conserved cysteine residues [51]. The linker region has a variable length and amino acid composition among family members and contains several sites vulnerable to protease degradation [51,52]. High-affinity IGF binding results from both the N- and C-terminus working together in concert, akin to a set of jaws that can contain the entire IGF molecule.

Little information is available concerning the exact role played by the IGFBP domain in CCN function. The IGFBP domain of the CCN proteins shares strong sequence similarity to the N-terminal domain of traditional IGFBPs [1] (Figure 1); this similarity has resulted in some CCN proteins being classified as additional IGFBPs or as IGFBP-related proteins (IGFBP-rPs) [51]. Despite the similarity with the N-terminal domains, the CCN proteins bind IGF poorly, in the order of 100-fold lower than the traditional IGFBPs. This low binding affinity probably results from the lack of an IGFBP C-terminal-like domain [53]. The construction of a recombinant chimera between CCN3 and IGFBP3 established that the CCN3 IGFBP module cannot substitute for the IFGBP3 amino-proximal sequence for IGF binding [54], an observation that reinforces the possibility that the IGFBP module of CCN proteins might be involved at another level of IGF signalling. It is possible that interactions with IGF could contribute to some types of CCN-dependent tumourigenesis.

**Figure 2:**
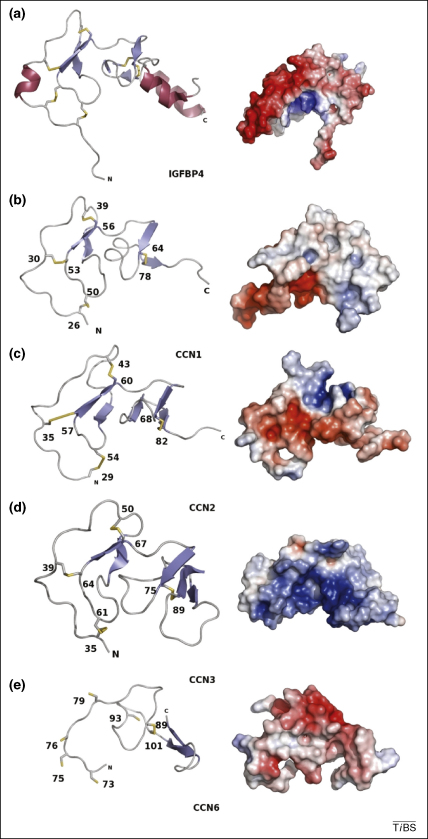
The IGFBP domain. The models of the IGFBP domain of (ii–iv) CCN1–3 and (v) CCN6 alongside (i) the IGFBP4 structure ([PDB code 2DSP) [55]. The models maintain the L-shaped structure with a disulphide supported ladder-like structure forming one lobe and a β-sheet-containing section forming the second lobe. In the surface representations of the IGFBP domains red represents negative charge and blue represents positive charge. The long protruding thumb region in IGFBP4 is clearly visible but could not be modelled for any of the CCN proteins. This figure was drawn using PyMOL (http://pymol.sourceforge.net/).

The close identity between the N-terminal domains of traditional IGFBPs and CCN proteins allows a structure to be predicted by the CPH model (World Wide Web Prediction) server*. The models were built using an ∼80 amino acid stretch of IGFBP4 with which they shared ∼30% sequence identity (PDB code 1DSP) [55]. The ∼80 residue IGFBP N-terminal domain has an approximately L-shaped appearance and can be divided into two subdomains connected by a short stretch of coil (Figure 2). The first subdomain has a novel fold with a two-stranded anti-parallel β-sheet and two parallel loops stabilized by a ladder of disulphide bonds [55]. The β-sheet and the disulphide ladder both form a flat plane, the so-called ‘palm’ of the molecule, and in the traditional IGFBPs, the N-terminal residues protrude forming a ‘thumb’ on the IGFBP-binding domain. This thumb plays an important role in IGF binding, forming several important hydrophobic interactions with aromatic residues within the IGF molecule [55]. The second subdomain contains the IGF-binding site and is a globular domain centred around a three-stranded anti-parallel β-sheet strengthened by an internal disulphide bridge that links strands 1 and 3 [55–57]. The palm, which contains the conserved GCGCCxxC motif, forms a rigid base that supports and separates the thumb sequence and the globular subdomain, maintaining the correct conformation to allow IGF binding [55].

**Figure 3:**
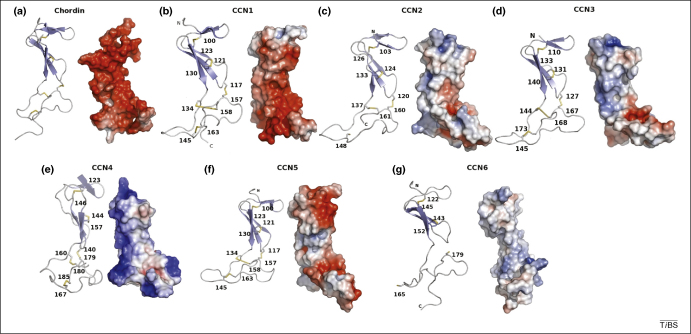
The VWC domain. The ∼70 residue stretch of the VWC domain from (ii–vii) the six CCN proteins alongside (i) the structure of the VWC domain from human chordin (PDB code IU5 M) [70] shown as ribbon and electrostatic surface models. The top half of the molecule with the two β-sheets is the first subdomain, and the lower half of the molecule held together only by disulphides is the part that resembles fibronectin and might be involved in growth factor binding. In the electrostatic surface representations of the VWC domains, positive charge is shaded blue and negative charge is shaded red. The differences in surface charge might contribute to the different biological functions observed for CCN family members. This figure was drawn using PyMOL (http://pymol.sourceforge.net/).

The IGFBP domain models generated by the CPH model server vary slightly among the four CCN proteins for which models were established (Figure 3); CCN4 and CCN5 could not be modelled. The models for CCN1, CCN2 and CCN3 resemble that of the IGFBP4 structure: they maintain the disulphide bonded ladder (containing three disulphide bonds) as well as a flat palm region and the cleft at the IGF-binding site. The CCN6 model, unlike the others, lacks the disulphide ladder and the flat palm region. However, this difference might be due to limitations with modelling and an inability to thread the first half of the IGFBP domain rather than a meaningful biological difference.

Notably, there are some differences between the CCN models and the IGFBP4 structure. For example, the N-terminal thumb region, which in IGFBP4 harbours an ‘XhhyC motif’, where ‘h’ is a hydrophobic amino acid, and ‘y’ is positively charged [55–57], is absent in CCN proteins. Although both CCN2 and CCN3 have positively charged residues directly preceding the cysteine, they have glutamine residues in place of one of the hydrophobic residues. The importance of this difference, if any, is unknown.

Although the models of CCN1–3 are all similar and share the same disulphide bonding pattern and general core structure, they display different surface electrostatic charges (Figure 3). As one of the underlying questions about the CCN proteins is how they interact with different binding partners while maintaining such close sequence and presumably structural similarity, subtle changes in the surface charge and the areas in which they interact with binding partners might allow CCN proteins to differentiate between their specific partners.

### The von Willebrand factor C repeat

The von Willebrand factor type C domain (VWC), also referred to as chordin-like cysteine rich (CR) repeats, is a common motif found in >500 ECM proteins [58]. This repeat is typically ∼70–100 amino acids in length and contains ten conserved cysteine residues and a pair of cysteine-containing motifs. The first of these, C_2_xxC_3_xC_4_, lies towards the middle of the repeat, and the second motif, C_8_C_9_xxC_10_, lies towards the end (in both cases the numbers refer to the ten cysteine residues that comprise the VWC repeat). In the CCN protein family, these motifs are conserved, but both motifs are extended with an extra residue between C_2_ and C_3_ and two extra residues between C_9_ and C_10_ [1] (Figure 1). However, four cysteine residues are missing (numbers 2, 6, 8 and 9) in CCN6 [1].

The VWC repeat is one of the most commonly occurring motifs and is found in many proteins of variable functions including the CCN proteins, procollagen, thrombospondin, von Willebrand factor, glycosylated mucins and neuralin [6]. Unlike the lone copy present in the CCN family of proteins, the VWC domain is typically present in multiple copies in other proteins [59,60]. In most proteins, the VWC repeat regulates bone morphogenic proteins (BMPs) and TGF-β signalling [59,61–63]. BMPs are important growth factors that influence bone and cartilage growth, skeletal patterning and the formation of several organs, such as the kidney, lungs and teeth [64,65]. The ability of the VWC domain to bind TGF-β and BMP suggests that it plays a role in some of the biological functions associated with CCN proteins. In proteins that contain multiple VWC domains, growth factor affinity varies among repeats, and the activity of intact proteins can be as much as tenfold higher than that for an individual repeat, suggesting a complicated means of regulation [66]. The significance of this effect on the function and regulatory activity of the single VWC domain in CCN proteins is unknown. However, it is likely that the sequence diversity among the VWC-containing proteins accounts for the variable substrate specificity. For example, CCN2 binds BMP4 and TGF-β1, although with a higher affinity for BMP4 (*K*_D_ 5 nM compared with 30 nM for TGF-β1) [59], whereas chordin binds BMP-4, -5 and -6 as well as TGF-β1 and TGF-β2 [62].

CCN2 might function as a chaperone for TGF-β1; therefore, the low-affinity binding might facilitate its transport to receptors that have affinities in the picomolar ranges [67]. The CCN2–TGF-β1 interaction also enhances TGF-β1 signalling, and less TGF-β1 is required to stimulate its downstream partners [59]. By contrast, CCN2–BMP4 binding is inhibitory [59]. Recent reports indicate that CCN3–BMP2 binding inhibits BMP2-induced osteoblast differentiation [68]. Related to its interaction with growth factors, the CCN3 VWC domain might participate in some aspects of cell development and tumour formation, perhaps by mediating its oligomerization. In von Willebrand factor, the VWC domain mediates large scale oligomerization, although only after an initial dimerization step has taken place. In CCN proteins, the CT domain has been implicated in dimerization, which could result in larger oligomers of CCN molecules through subsequent VWC domain interactions [4,5,69].

The VWC domains from CCN1–6 were modelled using the CPH model server^*^ using the NMR structure of a chordin VWC domain (PDB code IU5 M) [70] as the template. Aside from CCN6, each CCN protein contained the conserved cysteines of the domain. The chordin VWC domain forms two subdomains (Figure 3): the first comprises a short two-stranded anti-parallel β-sheet followed by a three-stranded anti-parallel β-sheet. The triple sheet is supported by a disulphide bridge between strands 2 and 3 and a second disulphide bond formed between strand 2 and the first strand of the two-stranded sheet. The second subdomain has no predicted secondary structure but is constrained by three disulphides formed between the remaining six cysteine residues. It forms a novel fold that shows some structural similarity to fibronectin. This finding could suggest that the interactions between the CCN proteins and TGF-β follow a similar pattern to that seen between VEGF and fibronectin [70]. Two regions in BMPs, a ‘knuckle’ and ‘wrist’, are important for receptor binding, and some results suggest that the negative activity that is elicited by VWC repeats might result from interference with the interactions between the ‘knuckle epitope’ and BMP-receptor II [70,71]. In considering the interactions between TGF-β, BMP-4 and the VWC domain in CCN proteins, it is impossible to predict the exact nature of the interaction or the location of the VWC domain until further biological/structural data become available.

**Figure 4:**
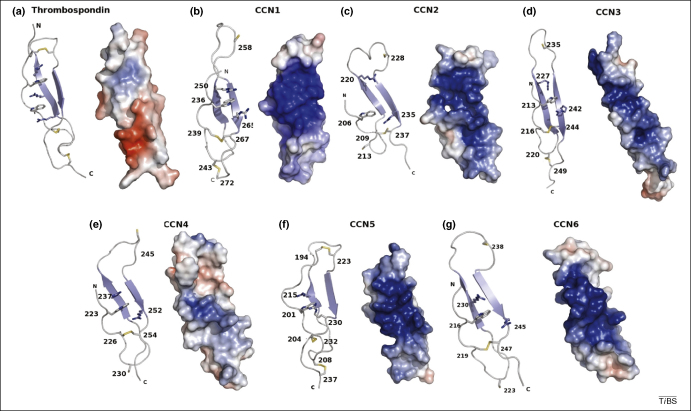
The CCN TSP domain. The ∼40 residue models of the TSP domain of (ii–vii) the CCN family shown alongside (i) the TSP-1 domain of thrombospondin (PDB code 1LSL) [84], shown as both ribbon models and electrostatic surface models. The two β-strands and the third unordered strand form the basis of the domain. Several residues that could form the ‘CWR’ layers are also shown in stick form. The first cysteine disulphide formation that forms the ‘top’ layer is not present in the model but is present in the protein sequence, and the unpaired half of it is shown at the top of the third strand. There are also two arginine residues and a tryptophan present that can form the CWR layers. The additional CWR layers in thrombospondin can also be observed. In the model structures of the TSP domains from the CCN family shown, positive charge is shaded blue and negative charge is shaded red. In each case, there is a large patch of positive charge that forms a groove that might permit interactions with heparin or other sulphated proteoglycans. This figure was drawn using PyMOL (http://pymol.sourceforge.net/).

The VWC domain in CCN proteins consists of an upper section comprising β-sheets and a more or less unstructured lower domain held together by disulphide bonds (Figure 3). The conserved cysteines in CCN1–5 all follow the same binding pattern: two disulphide bonds between the β-sheets and three more holding the unstructured fibronectin-like domain together [70]. Although each CCN protein is similar in VWC domain arrangement, their electrostatic surfaces (Figure 4) show a wide range of differences. CCN1 and to a lesser extent CCN5 are primarily negatively charged on the front face of the VWC domain, whereas CCN4 is primarily positively charged. The remaining molecules have a mix of charges on their surface. The large differences in surface charge could affect the different behaviours of CCN family members or their inter- or intra-molecular oligomerization patterns.

### The TSP-1 domain

The thrombospondin repeat domain is a ∼55 residue consensus sequence named for the ECM glycoprotein TSP-1 that contains repeats of three distinct domains within a primarily linear structure [72]. These include three thrombospondin type-1 repeats (TSR-1), three epidermal growth factor-like repeats (thrombospondin type-2 repeats) and seven aspartic-acid-rich repeats (thrombospondin type-3 repeats) [73].

The TSR-1 repeat is a common motif; 187 TSR sequences are found within the human genome and numerous others are found in other eukaryotic organisms [73]. TSR-1-containing molecules are wide ranging in scope and include, in addition to the CCN family, thrombospondins and spondins, papilin, ECM ADAMTS (a disintegrin and metalloproteinase with thrombospondin motif) and complement pathway proteins (C6, C7, C8A, C8B, C9 and properdin) [73,74]. Within this wide range of proteins, the TSP-1 domain binds many different targets, including collagen V [75], fibronectin [76], CD36 [77], TGF-**β** [78] and heparin [79], thereby eliciting many distinct biological functions. The functions of the thrombospondin TSR repeats have been extensively studied; four main functions are now attributed to them: (i) cell attachment sites in signalling and adhesion, (ii) inhibition of angiogenesis, (iii) protein-binding sites for a range of growth factors and other ECM proteins and (iv) glycosaminoglycan (GAG)-binding sites [80].

In the CCN family, the exact function of the TSP-1 domain remains unclear, although it is similar to TSRs in other proteins [37]. The ability of the TSP-1 domain to bind sulphated glycoconjugates [81] and some integrins [17] further suggests a role in adhesion or interactions with the ECM. As the CCN proteins are potent modulators of angiogenesis, it is also likely that the TSP-1 domain is an important module in the manipulation of angiogenesis. VEGF interacts with CCN2 through both the TSP-1 and CT domains [82]. The CCN2 TSP-1 domain interacts only with the heparin-binding VEGF-A_165_ isoform in an anti-angiogenic mode of action, whereas the CT domain interacts with both VEGF-A_165_ and VEGF-A_121_. The nature of the TSP–VEGF-A_165_ interaction suggests a mechanism through which the TSP-1 domain binds VEGF-A_165_ and sequesters it away from its receptors. This anti-angiogenic effect can also be removed after matrix metalloprotease (MMP)-mediated CCN2 cleavage [82,83].

Many TSRs bind TGF-β. For thrombospondin, this interaction requires the RFK tripeptide sequence located between the first and second TSRs. However, the TSP-1 domains in all of the CCN proteins lack this sequence. Therefore, another module might be involved with TGF-β binding [84], or the TSP domain might work synergistically with another of the TGF-β-binding domains.

On the sequence level, the TSP-1 domain shares a high degree of similarity with the other members of the TSR superfamily. It is a short domain of ∼60 amino acids with six conserved cysteine residues, the conserved motif CSxTCG (although CCN3 has the motif CSxSCG) and a commonly conserved arginine and a tryptophan residue at the N-terminal of the domain [1,84] (Figure 1).

The three-dimensional structures of the TSR repeats from thrombospondin [84] and F-spondin [85] have been determined by X-ray crystallography and nuclear magnetic resonance spectroscopy (NMR), respectively. Each individual TSR is composed of a small three-stranded anti-parallel β-sheet (∼15 × 20 × 55 Å) that is twisted slightly into a right-handed helical shape [84] (Figure 4). The three disulphide bonds present in each TSR repeat link the turns together to stabilize the structure; the disulphide bonding pattern can be used to divide the TSP family into two broad groups [84]. The network of bonds in the predicted CCN TSP-1 structures place them into group 2 alongside F-spondin, thrombospondin-related anonymous protein (TRAP) and the various proteins of the complement system [84]. Thrombospondin also contains a ladder-like arrangement of hydrogen bonds formed primarily by tryptophan and arginine residues, in conjunction with the supporting disulphide bonds, which give rise to the CWR layers, named after the residues that form the hydrogen bonds. The TSP domain in the CCN family lacks the WxxWxxW motif and has only one tryptophan. Similarly the CCN TSRs lack a pair of conserved arginine residues and, in most cases, contain a single arginine residue. This finding suggests that the CCN family TSP domains have fewer CWR layers criss-crossing the β-sheet. Although little is known about the binding properties of substrate to TSRs, they bind heparin, most likely through interactions with the negatively charged sulphate groups. In the structure of the thrombospondin TSR, the groove of the TSR helix is approximately the correct size for the accommodation of two heparin molecules and contains a large stretch of basic residues along the groove [84].

A predicted structure of the TSP-1 domains of all six CCN proteins was obtained using the CPH modelling server [55]; the predicted structure comprised ∼45 amino acids of the TSP domain out of the ∼70 amino acids in the domain (Figure 4). The models used were the malaria TRAP protein TSP domain (PDB code 2BBX) [85] for CCN1, 3, 5 and thrombospondin-1 (PDB code 1LSL) [84] for CCN2, 4 and 6 (based on which template produced the best model). All six models display the same spatial arrangement as existing TSP domains and have a three-stranded anti-parallel β-sheet. Strand A is irregular, but still forms a network of β-sheet-like bonds between strands B and C. However, unlike the structures of the F-spondin and thrombospondin TSRs, the predicted structure of the CCN TSP domains do not have such an extensive system of hydrogen bonds. In all six models, only a single tryptophan and two arginine residues are present. The three disulphide bonds that hold the loops together above and below the sheet are also present in the CCN domains, although in most of the models (except for CCN5), one or more are missing owing to the limitations of the modelled structure. Although the models do not show all of the disulphides, if they are extended to include the complete sequence of the TSP domain, the missing cysteines would be in the correct places (based on amino acid sequence alignment) to form the appropriate disulphide bonds. The electrostatic surfaces of the models (Figure 4) indicate that the strongly conserved basic face of the molecule probably interacts with heparin sulphate in a manner analogous to thrombospondin. Because the positively charged groove is conserved in all six of the models, it is possible that all of the CCN proteins use the TSP domain in a similar manner for binding heparin or sulphated proteoglycans to modulate cell adhesion and maintain ECM composition.

TSP domains interact with TGF-β [74], and, because interactions with TGF-β are central to many functions of CCN proteins, it is possible that these interactions might be coordinated by the TSP domain working in conjunction with the other CCN protein domains [3,4,8]. The TSP domain, like other CCN domains, might be involved in cancer biology [7]. Indeed, some studies have linked CCN proteins with mutant or missing TSP domains with colorectal and gastric carcinomas [6] and Wilm's tumours [86,87].

### The cysteine knot C-terminal domain

The CT domain is similar to those found in many extracellular matrix proteins, including *Drosophila melanogaster* slit, the von Willebrand factor, several mucins, Norrie disease protein and a wide variety of small growth factors, including VEGF, TGF-β, BMPs, nerve growth factor (NGF) and platelet-derived growth factor (PDGF) [1,88]. This domain is thought to mediate several CCN protein functions. Because it contains a cysteine knot motif of six conserved cysteine residues, it is thought to act as a dimerization module in a manner analogous to that seen in other growth factors, for example NGF, TGF-β or PDGF [1,4,89]. CT-domain-mediated interactions can be heterodimeric: the CCN3 and CCN2 CT domains interact in glutathione *S*-transferase pull-down assays [90]. CT-domain-mediated dimerization probably works in concert with TSP-domain-mediated oligomerization to give rise to the large CCN oligomers that have been detected in all preparations of CCN proteins so far described [37].

Several non-CCN protein CT domain interacting partners have been identified; these proteins might yield clues about CT domain function in CCN biology. Whereas the TSP domain is thought to bind heparin sulphated proteoglycans (HSPGs), the CT domain is involved in heparin binding. In addition, except for CCN4, which lacks the CT domain, the CCN proteins have a high number of basic residues at the CT domain N-terminus that follow the general heparin-binding pattern of xBBxBx (where B is a basic residue and x is usually not charged) [91]. The CT domain, in concert with the TSP domain, might be an important factor in directing how CCN proteins control and manipulate adhesion processes and the ECM composition and mediate cell adhesion [4]. Interactions with Notch 1, a key mediator of cellular differentiation [92], and the apoptosis-inducing integrin α_6_β_1_ [6] support a second role for the CT domain as a regulator of mitogenic signalling. Indeed, the isolated CCN2 CT domain performs this role [93]. The CCN3 CT-domain performs an essential role in the anti-proliferative function of CCN3, because in isolation this domain can prevent mesenchymal stem cell proliferation and differentiation [35]. The CCN3 CT domain is also thought to contain sequences that are involved in the nuclear addressing of CCN variants that lack a signal peptide [94]. Because nuclear CCN3 isoforms are potential negative regulators of transcription involved in tumourigenesis, the CT domain is an essential element that participates in the physiological balance between the distinct biological activities of intra- and extra-cellular CCN3 proteins.

The sheer number of biological functions tied to the heparin- and integrin-mediated receptors has resulted in suggestions that HSPGs and integrins should be considered as the functional receptors for the CCN family [12]. In addition to the pathways governed by HSPGs and integrins, the CT domain also modulates the Wnt signalling pathway through interactions with the low-density lipoprotein (LDL) receptor protein 6 (LRP6) [12]. Although incompletely characterized, each of these roles suggests that the CT domain is important for CCN function.

On the basis of its sequence, the CT domain is thought to be related to the ‘cysteine knot family’ of proteins, which encompasses the TGF-β-related growth factors and Norrie disease protein. Therefore, it is likely that it will interact with its partners in a similar manner [1,88]. The cysteine knot growth factors, although showing some sequence divergence, all share the same core three-dimensional structure [88]. This structure comprises an eight-membered ‘cysteine knot’ ring with two adjacent two-stranded anti-parallel β-strands and sometimes a short α-helix on the opposite side of the cysteine knot [88,89,95]. The cysteine knot is formed from a ring of eight residues linked by two disulphide bonds with a third bond through the centre of the knot [89,96]. In both NGF and TGF-β, the disulphide that forms through the knot is interchain, and a different cysteine directs dimer formation. By contrast, in PDGF, the cysteine coming through the centre of the knot forms an intrachain disulphide to complete the dimer. In addition to the ability to form strong dimers, TGF-β family members (including the BMPs) display high affinity for heparin and HSPGs [95,97]. The heparin-binding site occurs between the tips of the β-sheet loops and is located around a collection of positively charged residues. This binding site is primarily made up of four basic residues on the loop of the first β-sheet and then a single Arg/Lys on the loop of the second sheet [95,97]. Although this part of the sequence could not be modelled for all of the CCN proteins, they all possess this string of basic residues at the N-terminal portion of the CT domain.

**Figure 5:**
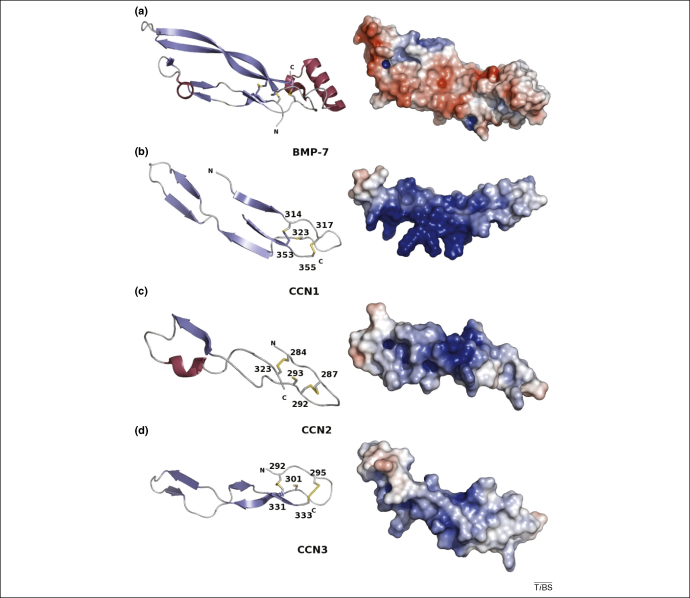
The CT domain. The ∼50 residue partial models of (ii–iv) the CT domains of CCN1–3 and (i) the structure of BMP7 (PDB code 1LXI) [100]. In each case the cysteine knot is visible with two disulphides forming the ring and a fifth cysteine protruding through the knot, available for either inter- or intra-molecular interactions. Electrostatic surface diagrams are also shown (red illustrates negative charge and blue illustrates positive charge) showing the subtle differences between the closely related molecules. This figure was drawn using PyMOL (http://pymol.sourceforge.net/).

The sequence divergence among the cysteine knot family could indicate why modelling the CT domain proved difficult (Figure 1). The Phyre homology and recognition server [98,99] could build only partial models of the CT domain for CCN1 using BMP7 as a template (PDB code 1LXI)] [100], CCN2 using TGF-β3 (PDB code 1KTZ) [101] and CCN3 using TGF-β1 as a template (PDB code 1KLA) [102]. In all cases, only ∼50 residue models could be constructed (Figure 5). In each case, the first ∼30 residues could not be modelled; these 30 residues include the basic N-terminal end of the domain and a long loop containing β-strands. However, the cysteine knot is intact with two disulphides forming the ring and a fifth cysteine protruding through the ring available for the formation of a third disulphide bridge. Without a complete three-dimensional structure, it is impossible to know whether this third disulphide will form an intra-molecular bond within the CT domain, or an inter-molecular bond and bind other cysteine-knot-containing growth factors, or a dimer with other CCN molecules [90]. Although the CCN CT domains appear to have a similar arrangement, the electrostatic surfaces (Figure 5) show some differences, and this finding, coupled with a fairly diverse sequence apart from the conserved cysteines, might account for the wide range in CT domain ligands and binding partners.

## Concluding remarks

CCN proteins participate in many cellular functions. Yet owing to the sheer scope of their action, it is unlikely that their function is a direct result of their individual domains acting independently, but rather represents a cumulative effect between multiple domains and receptors. Indeed the often opposing physiological functions of the structurally similar proteins supports this notion [3,4,11]. Although differences in amino acid sequence can lead to large variations in surface charge and substrate binding, the elucidation of the three-dimensional structure of individual domain or multiple domains is necessary to answer many remaining questions (Box 1) surrounding the CCN family of proteins.

An initial comparison of the primary structure of the six known CCN proteins could suggest that they possess redundant biological functions. However, the available experimental data strongly argue against that idea. The tight spatiotemporal regulation of the expression of CCN proteins [11] indicates that they are required at precise time frames. Interestingly, the expression patterns of CCN proteins show only partial overlap, which suggests that they might act sequentially. A thorough analysis of the regulatory processes involved in the coordinated expression of CCN genes is essential for a better understanding of the functions of the proteins they encode.

It has been proposed that the biological properties of CCN proteins are highly dependent upon the bioavailability of interacting ligands and proteins [11]. Accordingly, the tetramodular organization of the CCN proteins provides a unique situation, because each of the four constitutive modules could potentially interact independently with a handful of partners. Therefore, the variety of biological functions in which the CCN proteins participate can be viewed as the result of combinatorial events, the likelihood of which depends upon the presence of the various partners at precise times and locations [11]. Importantly, CCN proteins might act in concert and exert either synergistic or antagonistic functions. Indeed, recent research established that coordinated and interdependent CCN protein expression is required for chondrogenesis and osteogenesis [33,103].

Overall, the CCN proteins can be compared to non-identical ‘sextuplets’. Although they share many structural features, subtle differences in their interactions with binding partners render them functionally unique. The ability of CCN proteins to interact physically with various regulatory partners also places them at the interface of key signalling pathways. Whether the CCN proteins participate in the control and coordination of these signalling pathways and how this is achieved remain open and challenging questions.

## Figures and Tables

**Table 1:** Nomenclature of the CCN family of proteins^a^

CCN Family member	Alternative names
CCN1	Cyr61, CTGF-2, IGFBP10, IGFBP-rP4
CCN2	CTGF, IGFBP8, IGFBP-rP2, HBGF-0.8 ecogenin
CCN3	NOV, NOVH, IGFBP9, IGFBP-rP3
CCN4	Wisp-1, Elm-1
CCN5	Wisp-2, CTGF-L, CTGF-3, HICP, Cop-1
CCN6	Wisp-3

a Abbreviations: Cyr61, cysteine rich 61; CTGF-2, connective tissue growth factor 2; IGFBP-rP2, IGFBP-related protein 2; NOV, nephroblastoma overexpressed gene; HBGF-0.8, heparin-binding growth factor 0.8; Wisp, Wnt-inducible secreted protein; Elm-1, expressed in low metastatic cells; HICP, haparin-induced CCN-like protein; Cop-1 card-only protein 1.

## Box 1 Outstanding questions

(i) Could the differences in expression patterns, such as CCN1 and CCN2 being immediate early proteins, whereas CCN3 is not, or the differences in cellular locations and tissue specificity of CCN proteins provide some explanation for their apparently conflicting functions?
(ii) Does the presence of multiple binding sites spread over several domains and the altered biological activity of truncated CCN proteins support a synergistic inter-domain approach to substrate binding and function?
(iii) As the CT domain can form both homo- and hetero-dimers and the VWC domain might facilitate the formation of higher order complexes, could some CCN protein functions be orchestrated by larger complexes of CCN molecules?
(iv) Are the linker regions important for the biology of CCN proteins, either through proteolytic control or flexibility regarding hinges, thereby allowing distant domains to interact with each other?

## Glossary

ADAMTS: a disintegrin and metalloproteinase that harbours a thrombospondin motif.
CCN: the CCN family of proteins is a complex family of multifunctional proteins with six members designated CCN1 to CCN6. The CCN acronym was introduced in reference to the names of the first three members of the family to be discovered: Cyr61 (cysteine-rich protein 61), CTGF (connective tissue growth factor) and NOV (nephroblastoma overexpressed gene).
CT: C-terminal cysteine knot domain. This domain has a conserved pattern of cysteines consistent with a six membered cysteine knot similar to several members of the TGF-β growth factor family.
HSPGs: heparin sulphated proteoglycans – multifunctional molecules involved in cell and protein adhesion and fine tuning regulatory proteins.
IGFBP: the insulin-like growth factor binding protein (IGFBP) family comprises six IGFBPs (1–6) that bind insulin-like growth factors (IGFs) with high affinity (K_D_ ∼0.1 nM). IGFBPs exert their biological effects by modulating IGF behaviour.
SMAD: small mothers against decapentaplegic – a family of transcription factors that can modulate TGF-β behaviour.
TSP: the thrombospondin repeat domain is a ∼55 residue consensus sequence named after the extracellular matrix glycoprotein thrombospondin-1 (TSP-1) that contains repeats of three distinct domains within a primarily linear structure. The TSP-1 domain binds a wide range of extracellular targets and important signalling molecules including collagen V, fibronectin, TGF-β and heparin.
VWC: von Willebrand factor C repeat domains, also referred to as chordin-like cysteine-rich (CR) repeats, are an extremely common motif found in >500 extracellular matrix proteins. This repeat typically comprises ∼70–100 amino acids with ten conserved cysteine residues and a pair of cysteine-containing motifs located in the sequence.

## Footnotes

* O. Lund *et al.*, abstract A102, CASP5 (the 5th community-wide experiment on the Critical Assessment of Techniques for Protein Structure Prediction) conference, California (USA), December 2002.
